# Characterization and phylogenetic analysis of *Corydoras trilineatus* mitochondrial genome

**DOI:** 10.1080/23802359.2020.1797551

**Published:** 2020-07-30

**Authors:** Lei Chen, Baohong Xu, Tiaoyi Xiao, Qiaolin Liu

**Affiliations:** a Hunan Engineering Technology Research Center of Featured Aquatic Resources Utilization, Hunan Agricultural University, Changsha, PR China; b Collaborative Innovation Center for Efficient and Health Production of Fisheries in Hunan Province, Changde, PR China

**Keywords:** *Corydoras trilineatus*, mitochondrial genome, next-generation sequencing, phylogenetic analysis

## Abstract

In this study, the complete mitochondrial genome yielded by next-generation sequencing of *C. trilineatus* was assembled and analyzed. The total length of the mitochondrial genome is 16,526 bp. It contains two ribosomal RNA genes, 13 protein-coding genes, 22 transfer RNA genes, and a major non-coding control region . The arrangement of these genes is the same as that found in the Siluriformes. The complete mitogenomes of *C. trilineatus* and other 17 species from nine genera were used for phylogenetic analysis by UPGMA method. The topology demonstrated that all species are divided into three groups , and the *C. trilineatus* was clustered with *C. rabauti* and *C. nattereri*.

‘Mouse fish’ contains the species from the genera *Aspidoras*, *Brochis*, and *Corydoras* belonging to subfamily Corydoradinae. As there are two small beards next to the mouth, which is like a little mouse swimming in the water, it is called ‘Mouse fish’. Three-lined Corydoras (*Corydoras trilineatus*) is a kind of famous ornamental mouse fish. It has a light straw body with black bands and brown edges on its sides. There is a very black spot on the dorsal fin and five vertical stripes on the tail. It is probably the most misidentified fish for *C. julii*. In fact, it is mid-lateral black stripes fainter and does not reach as far toward the fish head as in *C. trilineatus*.

In order to distinguish and identify the species more accurately, we determined the mitochondrial genome sequence of the *C. trilineatus* using next-generation sequencing technology. Also mitochondrial genome structure and phylogeny were analyzed. The living body of *C. trilineatus* was collected from the Red Star Ornamental Fish Market in Changsha, Hunan Province, China (113.03 E, 28.09 N). After anesthesia with MS-222 (3-aminobenzoic acid ethyl ester methanesulfonate), dorsal muscle tissue named ML004 was collected and preserved in 99% ethanol in Museum of Hunan Agricultural University. After DNA extraction (Tissue DNA Kit D3396-02, Omega, Bio-Tek, Norcross, GA) and sequencing library construction (Sangon Biotech, Shanghai, China), paired end reads were sequenced using HiSeq XTen PE 150 of Illumina (San Diego, CA). BBduk and BLAST + were used to assess and monitor data quality. NOVOPlasty and SPAdes were used for *de novo* assembly. MITOS2 server and Geneious R11 (Liu et al. 2019; Tan et al. 2019) were used to predict and annotate the mitochondrial genome. Geneious Tree Builder was utilized to build UPGMA phylogenetic tree with Tamura-Nei (genetic distance model).

Totally 22,270,672 high-quality clean reads (150 bp PE read length) were obtained. The total length of the mitochondrial genome is 16,526 bp (GenBank accession number: MT478052), with the base composition of 32.59% A, 14.92% G, 26.35% T, and 26.15% C, with a slight A + T bias of 58.94%. It contains two ribosomal RNA genes, 13 protein-coding genes, 22 transfer RNA genes, and a major non-coding control region (D-loop region). Most of the genes were encoded on the heavy strand except ND6 and seven tRNA genes (tRNAGln, tRNAAla, tRNACys, tRNATyr, tRNASer, tRNAGlu, and tRNAPro) encoded on the light strand. The arrangement of these genes is the same as that found in the Siluriformes (Saitoh et al. 2003; Liu et al. 2019). All the protein initiation codons are ATG, except for cox1 that begins with GTG. The complete mitogenomes of *C. trilineatus* and other 17 species from nine genera were used for phylogenetic analysis by UPGMA method. The topology demonstrated that all species are divided into three groups (Siluridae, Callichthyidae, and Loricariidae), and the *C. trilineatus* was clustered with *C. rabauti* and *C. nattereri* (Figure 1). The information of the mitogenome provides a basis for future phylogenetic studies and species identification of *Corydoras*.

**Figure 1.**
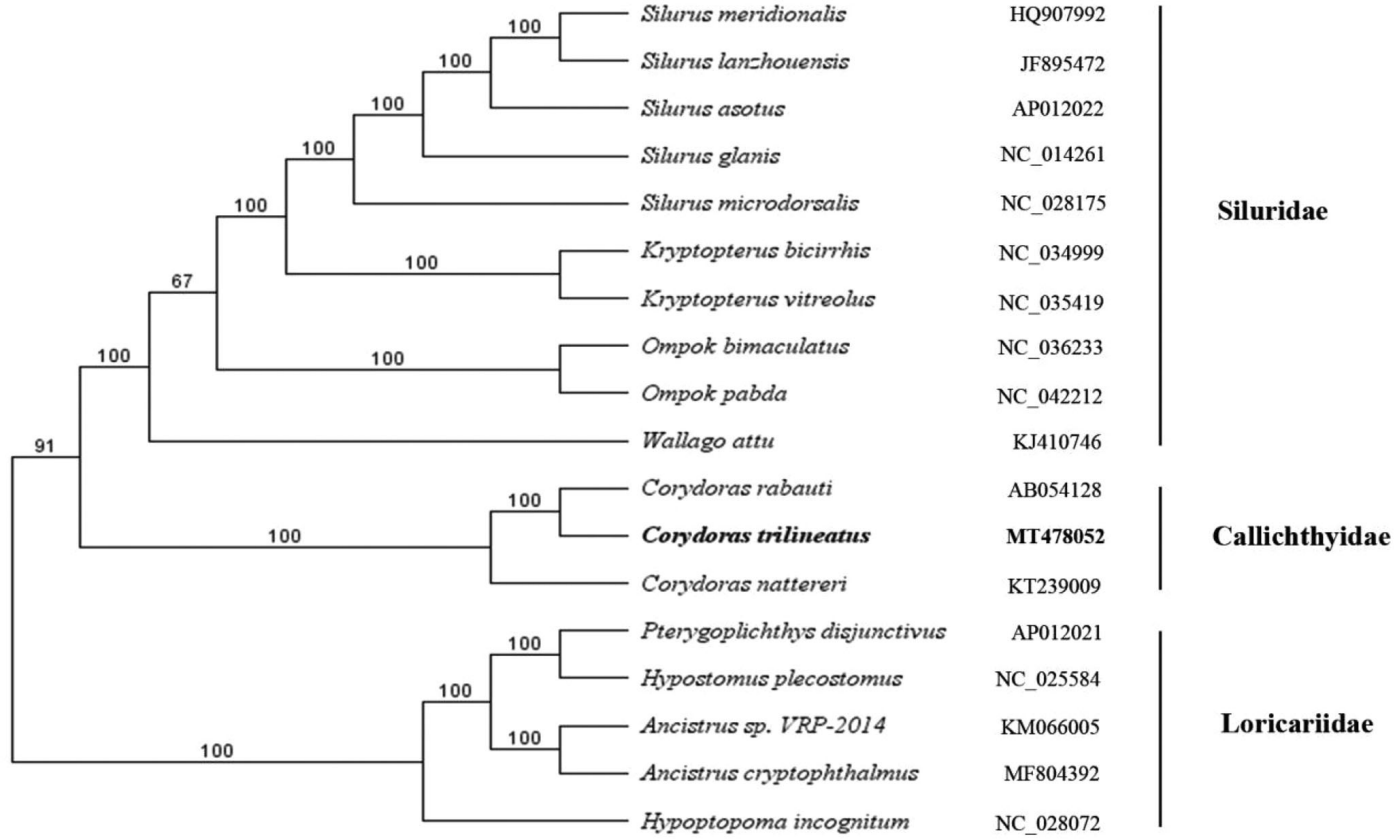
UPGMA phylogenetic tree based on the complete mitochondrial genome sequence. Note the bold Latin name represents the species in this study. The codes followed the Latin names were GenBank accession numbers for each mitogenomes.

## Data Availability

The data that support the findings of this study are openly available in GenBank, reference number [GenBank accession number: MT478052], URL [https://www.ncbi.nlm.nih.gov/nuccore/MT478052.1/].
